# Accuracy of ECG chest electrode placements by paramedics: an observational study

**DOI:** 10.29045/14784726.2021.6.6.1.8

**Published:** 2021-05-01

**Authors:** Pete Gregory, Tim Kilner, Stephen Lodge, Suzy Paget

**Affiliations:** University of Wolverhampton ORCID iD: https://orcid.org/0000-0001-9845-0920; University of Worcester ORCID iD: https://orcid.org/0000-0001-7725-4402; University of Wolverhampton; University of Wolverhampton

**Keywords:** ECG, ECG training, paramedics

## Abstract

**Background:**

The use of the 12-lead electrocardiogram (ECG) is common in sophisticated pre-hospital emergency medical services but its value depends upon accurate placement of the ECG electrodes. Several studies have shown widespread variation in the placement of chest electrodes by other health professionals but no studies have addressed the accuracy of paramedics. The main objective of this study was to ascertain the accuracy of the chest lead placements by registered paramedics.

**Methods:**

Registered paramedics who attended the Emergency Services Show in Birmingham in September 2018 were invited to participate in this observational study. Participants were asked to place the chest electrodes on a male model in accordance with their current practice. Correct positioning was determined against the Society for Cardiological Science and Technology’s 2017 clinical guidelines for recording a standard 12-lead ECG, with a tolerance of 19 mm being deemed acceptable based upon previous studies.

**Results:**

Fifty-two eligible participants completed the study. Measurement of electrode placement in the vertical and horizontal planes showed a high level of inaccuracy, with 3/52 (5.8%) participants able to accurately place all chest electrodes. In leads V_1_–V_3_, the majority of incorrect placements were related to vertical displacement, with most participants able to identify the correct horizontal position. In V_4_, the tendency was to place the electrode too low and to the left of the pre-determined position, while V_5_ tended to be below the expected positioning but in the correct horizontal alignment. There was a less defined pattern of error in V_6_, although vertical displacement was more likely than horizontal displacement.

**Conclusions:**

Our study identified a high level of variation in the placement of chest ECG electrodes, which could alter the morphology of the ECG. Correct placement of V_1_ improved placement of other electrodes. Improved initial and refresher training should focus on identification of landmarks and correct placement of V_1_.

## Introduction

International guidelines for the management of patients presenting with symptoms suggestive of an acute coronary syndrome recommend that a 12-lead electrocardiogram (ECG) be recorded by attending Emergency Medical Service (EMS) personnel prior to hospital conveyance (Garvey et al., 2006; Ibanez et al., 2018; O’Gara et al., 2013; Ting et al., 2008). The recording of a pre-hospital ECG has become increasingly common in sophisticated pre-hospital EMS systems and has been shown to significantly increase the proportion of patients who receive primary percutaneous coronary intervention (pPCI) within 90 minutes of calling the EMS, and to increase the number of ST-elevation myocardial infarction (STEMI) patients who receive fibrinolytics in hospital within 30 minutes of arrival (Quinn et al., 2014). Patients who receive a pre-hospital ECG also exhibit significantly lower hospital and 30-day mortality rates than those who do not, with most of the differences attributable to significantly lower rates of mortality in STEMI patients (Quinn et al., 2014). However, the patient benefit that can be derived from the pre-hospital recording of a 12-lead ECG is reliant upon the ability of EMS personnel to recognise STEMI, or to have access to telemetry to allow another healthcare professional to make the decision, and to accurately place the ECG electrodes. Studies have investigated the ability of EMS personnel to interpret 12-lead ECG recordings in cases of STEMI (Cantor et al., 2012; Mencl et al., 2013; O’Donnell et al., 2015; Whitbread et al., 2002), but none have explored the ability of EMS personnel to correctly place the electrodes.

Incorrect positioning of precordial electrodes presents a risk to patients as it can lead to morphological changes in the ECG (Bond et al., 2012; Kania et al., 2014; Walsh, 2018), with subsequent misinterpretation. The risks are as yet unquantified but there is potential for a patient to receive harmful therapeutic procedures or to encounter a delay in the administration of, or potentially the withholding of, beneficial therapeutic procedures. Studies in Europe, North America and Australia have investigated the accuracy of precordial electrode placement with other health professionals and have highlighted varying degrees of accuracy. Rajaganeshan et al. (2008) found that the correct position for V_1_ was identified by 90% of cardiac technicians, 49% of nurses, 31% of physicians (excluding cardiologists) and only 16% of cardiologists. This study also saw a frequent malposition of V_5_ and V_6_. Medani et al. (2018) found that only 10% of participants (doctors, nurses and cardiac technicians) correctly applied all of the leads, with the most common errors being the placement of the V_1_ and V_2_ leads too superiorly and the V_5_ and V_6_ leads too medially. McCann et al. (2007) found clinically significant variability in the identification of standardised precordial electrode positions among senior emergency clinicians. An older American study (Wenger & Kligfield, 1996) found that leads V_1_ and V_2_ were commonly placed superior and lateral of the anatomical location, and that electrodes V_4_–V_6_ were commonly placed inferior and lateral of the specified point. From these studies, we hypothesised that there was likely to be a high level of inaccuracy in the placement of the precordial electrodes by EMS personnel.

The primary objective of this prospective observational cohort study was to identify the accuracy of precordial electrode placement by UK registered paramedics. We opted not to look at limb leads at this stage, although we acknowledge that incorrect placement of limb leads may occur and may affect the accuracy of the reading.

## Methods

Participants were recruited at the Emergency Services Show in Birmingham, UK on 19–20 September 2018. Participants were eligible if they were on the Health and Care Professions Council register (paramedic) at the time of the study, and trained and authorised to record and interpret 12-lead ECGs in the out-of-hospital setting. Recruitment was through posters displayed at the show, promotion by the College of Paramedics (UK professional body) at their seminar sessions and word of mouth at the show. Participants were provided with an information sheet and a briefing from the researcher, with an opportunity to ask questions. Written informed consent was obtained from all participants before data were collected. Data were anonymised and information on the performance of individual participants was not made available to anybody outside the research team. Participants did not receive any reward for their participation.

Participants provided professional demographic information relating to their length of experience as a paramedic, the recency of their practice, their academic route to qualification (university route or vocational route), whether they had a specialist role and the time since their last formal training on ECG electrode placement. Information was collected electronically through the Jisc Online Survey tool (https://www.onlinesurveys.ac.uk/), which allocated a unique identifier to each participant and removed the need to collect person identifiable information. Participants were then asked to place the six precordial electrodes on to the chest of a human male model in accordance with their current practice. The model was an adult male in his mid-20s with easily defined landmarks and a non-hairy chest. The specific model was chosen as we wanted to control for other factors that could cause incorrect electrode placement, such as breast tissue. He was placed on an examination couch inclined to 45° and was undressed to the waist for the procedure. For purposes of privacy and minimising distraction, the model was concealed from onlookers by screens. Neither the participants nor the model received any reward, monetary or otherwise, for their participation in the study.

Before measurement, participants were asked to confirm that they were satisfied with their positioning and were offered an opportunity to make an adjustment if they felt it necessary.

Prior to participant enrolment, the correct placements had been pre-determined by two paramedics and an advanced clinical practitioner in accordance with the Society for Cardiological Science and Technology’s 2017 clinical guidelines for recording a standard 12-lead ECG (Campbell et al., 2017). To maximise the accuracy of our electrode placement, we followed precisely the guidelines, measured the mid-clavicular point with a tape measure for V_4_ accuracy and had confirmation from an advanced clinical practitioner who was not directly involved with the study. We used a transparent overlay sheet to mark the exact position of our electrodes. The overlay was attached to the model using Transpore™ tape and the position of the corners was marked on the model’s chest using a fine marker pen. The corners of the overlay could then be re-located against the marks and, for consistency, the same researchers placed the overlay into position and completed the measurements. The overlay was pre-printed with 5 mm boxes to assist with the visualisation of the measurement. We used Skintact^®^ FS50C electrodes as they were typical electrodes for ambulance service use and had a centrally placed connector which was used as a consistent measuring point. Deviation from our positioning was recorded in the vertical and horizontal planes, with a deviation of 19 mm deemed to be within an acceptable tolerance. This was based on a previous study by Kania et al. (2014) which demonstrated that more prominent morphology changes of ECG waves were found for electrode displacements of 2 cm or greater. Data were input into Microsoft^®^ Excel and then plotted on a scatter graph to show dispersal from the centre point of our electrode.

Electrode placement was noted in distance (mm) from the reference point in both the vertical and horizontal planes. Data were analysed using SPSS Statistics for Macintosh (Version 26.0, Armonk, NY). Given the small sample size, normality of distribution of the data was assessed using the Shapiro-Wilk test. The data relating to the vertical plain were determined to be normally distributed while the data relating to the horizontal plane were not normally distributed.

Correlation between electrode placements (relative to each other) in the vertical plane was analysed by way of parametric testing, specifically Pearson correlation. Analysis of correlation between electrode placements relative to each other in the horizontal plane required non-parametric testing and was conducted using Spearman’s correlation. Significance was accepted as p < 0.05 for both datasets. In line with normal convention, measures of central tendency and dispersion are reported as mean with standard deviation (SD) for the normally distributed data (vertical plane) and median with interquartile range (IQR) for the non-normally distributed data (horizontal plane).

### Patient and public involvement

There was no patient or public involvement in this study.

## Results

Fifty-two eligible participants completed the study, the characteristics of whom are summarised in Table 1. The majority of participants had taken a higher education route to paramedic registration, although a small number had gained registration through the legacy vocational training routes. All those included in our sample were trained and working in the UK. There was a wide variation in the time since many participants had received training in ECG electrode placement, with a range from less than six months to more than five years.

**Table 1.** Participant characteristics.

**Specialist role**	**Number (%)**
Primary care	7 (13.5)
Critical care	1 (1.9)
Training officer	1 (1.9)
**Years of whole time equivalent as paramedic**	
0–4	31 (62.0)
5–9	7 (14.0)
> 10	12 (24.0)
**Currency of practice**	
Current	45 (86.5)
Within last 12 months	0 (0.0)
Between 1 and 5 years ago	4 (7.7)
More than 5 years ago	3 (5.8)
**Educational route to registration**	
IHCD (vocational training)	8 (15.4)
Certificate of higher education	1 (1.9)
Diploma of higher education/Foundation degree	37 (71.2)
BSc/BSc (Hons)	6 (11.5)
**Higher degree in clinical practice (Master’s or doctorate)**	
Yes	4 (7.7)
No	48 (92.3)
**Time since last formal ECG training**	
Within last 6 months	3 (5.8)
Between 6 months and 1 year	10 (19.2)
1–2 years	11 (21.2)
2–5 years	12 (23.1)
> 5 years	16 (30.8)

ECG = electrocardiogram; IHCD = Institute of Health and Care Development.

The positioning of the ECG electrode was analysed in respect of the vertical and horizontal planes relative to the pre-determined reference position. The data relating to the vertical plain were determined to be normally distributed, while the data relating to the horizontal plane were not normally distributed. Table 2 illustrates the mean and SD for the normal data in the vertical plane, and the median and IQR for non-normal data of the horizontal plane. Only three participants were able to correctly place all leads.

**Table 2.** Average distances (in mm) from correct placement in vertical and horizontal planes.

Vertical plane	Mean (SD)	Horizontal plane	Median (IQR)
V_1_	12.94 (18.42)	V_1_	13 (12)
V_2_	19.75 (19.82)	V_2_	15 (11)
V_3_	-8.85 (20.33)	V_3_	7 (12)
V_4_	19.48 (17.23)	V_4_	17 (19)
V_5_	-18.12 (18.83)	V_5_	0 (23)
V_6_	13.69 (21.29)	V_6_	0 (18)

IQR = interquartile range; SD = standard deviation.

**Figure 1:**
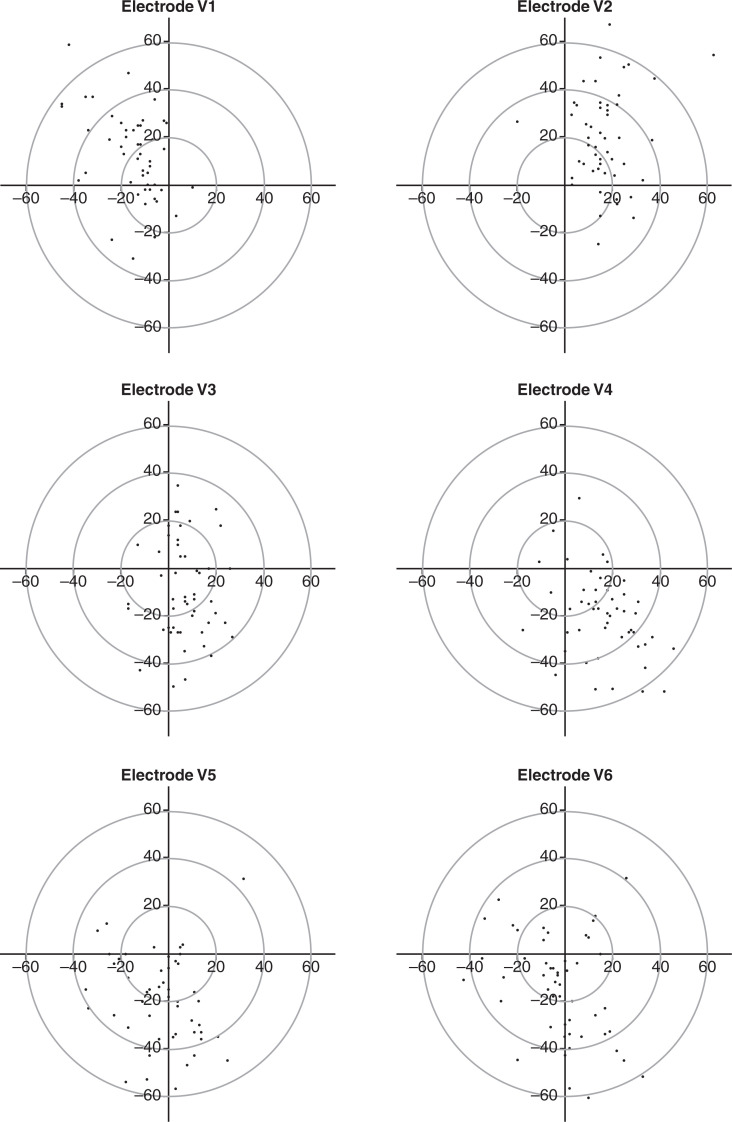
Figure 1. Scattergram of electrode placements by lead.

The positions of the electrodes are shown in Figure 1. There was substantial variation in the positioning of all electrodes, with patterns of incorrect displacement emerging in V_1_–V_5_. In V_1_ and V_2_, the majority of errors were related to the electrodes being positioned too high on the chest. The majority (75% for V_1_ and 67% for V_2_) were able to place the electrode correctly on the horizontal plane. The highest displacement for both V_1_ and V_2_ would have placed the electrode in the second intercostal space.

In V_3_, the majority of incorrect placements were related to vertical displacement, with most participants (87%) able to identify the correct horizontal position. In V_4_, the tendency was to place the electrode too low and to the left of the pre-determined position, with only one placement being displaced too high. Placement of V_5_ tended to be below the expected positioning, although 77% were able to correctly identify the correct horizontal placement. There was a less defined pattern of error in V_6_, although vertical displacement was more likely than horizontal displacement in terms of absolute numbers and degree of error.

Further analysis of data sought to establish correlation between the placement of electrodes across vertical and horizontal planes. A two-tailed Pearson bivariate correlation was undertaken; these are presented in Table 3.

**Table 3.** Correlation between electrode placements (two-tailed) in vertical and horizontal planes.

**Pearson correlation coefficient (vertical plane)**						
V_1_		.962*	.692*	.348*	.184	.181
V_2_	.962*		.677*	.283*	.203	.182
V_3_	.692*	.677*		.636*	.375*	.295*
V_4_	.348*	.283*	.636*		.607*	.547*
V_5_	.184	.203	.375*	.607*		.900*
V_6_	.181	.187	.295*	.547*	.900*	
**Spearman correlation coefficient (horizontal plane)**						
	V_1_	V_2_	V_3_	V_4_	V_5_	V_6_
V_1_		-.117*	.042	.070	.093	.159
V_2_	-.117*		.372*	.324*	.421*	.413*
V_3_	-.042	.372*		.548*	.377*	.125
V_4_	.070	.324*	.548*		.713*	.358*
V_5_	.093	.421*	.377*	.713*		.804*
V_6_	.159	.413*	.125	.358*	.804*	

* = statistically significant at p ≤ 0.05.

## Discussion

In this study, we found significant variation in the placement of the chest electrodes by registered paramedics. Incorrect positioning of electrodes has been well established as a cause of artefact on the ECG (Bond et al., 2012; Harrigan et al., 2012; Kania et al., 2014; Rudiger et al., 2007; Walsh, 2018), which poses risks to the patient. Patients may receive treatment that is potentially harmful and unnecessary, or they may have appropriate treatment withheld; there is the additional risk of conveyance to a unit without pPCI capability, therefore delaying this treatment, or possibly conveying to a pPCI centre where no emergency department exists when the patient is not indicated for a pPCI centre. In addition, there is the potential danger created by inappropriate transport under emergency conditions. Correct placement of ECG electrodes is also important for reproducibility and diagnosis where serial comparison is undertaken.

Previous studies with other health professionals have identified common misplacement of leads V_1_ and V_2_ (Rajaganeshan et al., 2008; Walsh, 2018), with a similar pattern reflected in our study. Placement of both of these leads tended to be significantly higher than the recommended placement, with many electrodes situated within the second or third intercostal space. Walsh (2018) has demonstrated that the ECG resulting from such misplacement may generate erroneous patterns such as incomplete right bundle branch block, anterior T wave inversion, septal Q waves or ST-segment elevation.

The identification of anatomical landmarks is important for the correct placement of electrodes but many participants in our study did not seek to formally identify these landmarks. This meant that when V_1_ was incorrectly placed, V_2_ would be incorrectly placed in a mirror image. The correlation shown between electrodes V_1_ and V_2_ is suggestive that electrode placements were influenced by previous electrode location rather than identification of anatomical landmarks. For electrodes V_2_, V_3_ and V_4_, it would be expected that a high positive correlation would exist given that V_3_ is positioned midway between V_2_ and V_4_. This was the case in the vertical plane, although the relationship between electrodes was not as strong as would have been expected; the reason for this is unclear. As V_2_ was incorrectly placed in a high number of cases in our study, it follows that V_3_ was also misplaced. Electrodes V_4_–V_6_ should be placed at the same horizontal level, so again a high correlation would be expected in the vertical plane. Correlation was strong in these electrodes, but this led to propagation of inaccuracy as misplacement of one electrode influenced misplacement of subsequent electrodes.

We carefully considered our choice of model, as other studies have identified obesity and modesty in females as factors linked with poor chest electrode placement (MacAlpin, 2017; Walsh, 2018). We also ensured that the conditions for the study were optimal in order to minimise extraneous factors that could affect performance. The process did not involve removal or displacement of clothing; the patient was well and therefore there was no stress involved; and participants were not being observed by other conference participants. Our chosen model was a male subject of medium build with easily identifiable landmarks, so did not present with the complexities of female or obese patients; it is postulated that our results would have revealed greater placement inaccuracy in a less-controlled environment and had our model been overweight or female.

Our sample size was relatively small and self-selecting, which will impact the generalisability of the results; however, our findings are similar to those from previous studies involving other health professionals (McCann et al., 2007; Medani et al., 2018; Rajaganeshan et al., 2008) and it does suggest a pattern of inaccuracy that causes concern. It could be argued that participants who attend a professional exhibition and conference may be more motivated than the wider paramedic population and if this hypothesis is accepted, it is likely that the accuracy of electrode placement in the wider paramedic profession will be less accurate than in our study population. We have established that correct placement of V_1_ increases the likelihood that other electrodes will be correctly placed, so we would recommend that educators become aware of this and focus on ensuring that V_1_ is correctly placed. From a patient safety perspective, we would also advocate that paramedics leave the chest electrodes in situ where manufacturer recommendations permit; this will allow hospital clinicians and/or ECG technicians to assess the accuracy of the placement and either utilise the same positioning for a comparative ECG recording, or disregard the findings of the pre-hospital ECG.

## Conclusion

Our study identified a high level of variation in the placement of chest ECG electrodes by UK registered paramedics. It is not known to what extent, if any, incorrect placement has resulted in incorrect ECG interpretation or patient management, but the inaccuracy by our study participants was high and likely to cause morphological changes that could impact on patient treatment. It also raises questions as to the reliability and replication of findings of ECGs from patient to patient as well as for serial recordings over time for any given patient. We would argue that there is a need for improved initial training for paramedics and also for more frequent refresher training that emphasises the need to measure landmarks in order to ensure correct electrode placement. Our work also identified that if the paramedic places V_1_ correctly, they are more likely to place the others correctly; this is an important consideration for those teaching electrode placement, and educators need to be aware of the importance of this during initial and refresher training.

### Limitations

Our sample size was small and was recruited through a convenience sampling strategy. It is possible that the sample may not be reflective of the wider paramedic population in the UK or internationally, but the results do reflect patterns of inaccuracy that have previously been identified in studies involving other health professionals.

## Author contributions

In accordance with ICMJE guidelines, I can confirm that all authors meet the following criteria for authorship: substantial contributions to the conception or design of the work; or the acquisition, analysis or interpretation of data for the work; AND drafting the work or revising it critically for important intellectual content; AND final approval of the version to be published; AND agreement to be accountable for all aspects of the work in ensuring that questions related to the accuracy or integrity of any part of the work are appropriately investigated and resolved. PG acts as the guarantor for this article.

## Conflict of interest

None declared.

## Ethics

Ethics approval was obtained from the University of Wolverhampton Research Ethics Committee.

## Funding

None.
